# Insights on the Functional Interaction between Group 1 Metabotropic Glutamate Receptors (mGluRI) and ErbB Receptors

**DOI:** 10.3390/ijms21217913

**Published:** 2020-10-24

**Authors:** Ada Ledonne, Nicola B. Mercuri

**Affiliations:** 1 Department of Experimental Neuroscience, IRCCS Fondazione Santa Lucia, 00143 Rome, Italy; 2 Department of Systems Medicine, Università di Roma “Tor Vergata”, 00133 Rome, Italy; mercurin@med.uniroma2.it

**Keywords:** group 1 metabotropic glutamate receptors (mGluRI), mGluR1, mGluR5, ErbB receptors, tyrosine kinases, neuregulins, glutamate

## Abstract

It is well-appreciated that phosphorylation is an essential post-translational mechanism of regulation for several proteins, including group 1 metabotropic glutamate receptors (mGluRI), mGluR1, and mGluR5 subtypes. While contributions of various serine/threonine protein kinases on mGluRI modulation have been recognized, the functional role of tyrosine kinases (TKs) is less acknowledged. Here, while describing current evidence supporting that mGluRI are targets of TKs, we mainly focus on the modulatory roles of the ErbB tyrosine kinases receptors—activated by the neurotrophic factors neuregulins (NRGs)—on mGluRI function. Available evidence suggests that mGluRI activity is tightly dependent on ErbB signaling, and that ErbB’s modulation profoundly influences mGluRI-dependent effects on neurotransmission, neuronal excitability, synaptic plasticity, and learning and memory processes.

## 1. Group 1 Metabotropic Glutamate Receptors (mGluRI)

Group 1 metabotropic glutamate receptors (mGluRI) are G protein-coupled receptors (GPCR) comprising two closely related subtypes: mGluR1 (GRM1) and mGluR5 (GRM5). mGluR1 exist in four isoforms (mGluR1α, β, γ, δ) whereas mGluR5 exist in three variants (mGluR5a, b, d); different isoforms are produced by alternative genetic splicing and mainly differ for C-terminal intracellular tail [1,2,3]. Distinction from other mGluR subgroups (i.e., group II, including mGluR2 and mGluR3, and group III, comprising mGluR4, mGluR6, mGluR7, mGluR8) is based on amino-acid homology, agonist binding, and signaling pathways downstream to receptor activation [1,2,3].

**Figure 1:**
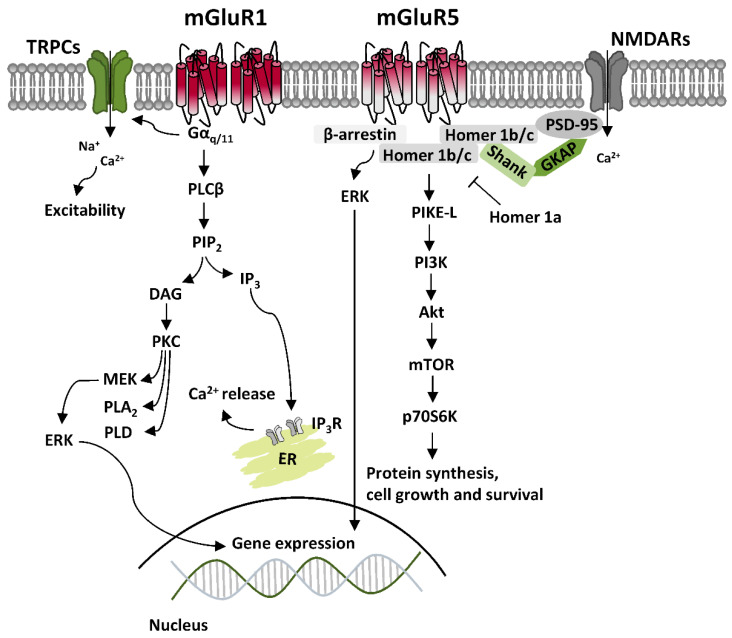
Group 1 metabotropic glutamate receptors (mGluRI) signaling. Scheme of the principal signaling pathways downstream to mGluR1 and mGluR5, showing G_q/11_-dependent activation of phospholipase C β (PLCβ), which mediates the hydrolysis of phosphatidylinositol and generation of inositol-1,4,5-trisphosphate (IP_3_) and diacylglycerol (DAG), that fosters Ca^2+^ intracellular mobilization from internal stores. Protein kinase C (PKC), also activated by DAG, is involved in the stimulation of extracellular signal-regulated kinase (ERK), affecting gene expression. mGluR1/5, through G_q/11-_dependent mechanisms, also induce the opening of transient receptor potential channels (TRPCs), mainly responsible for mGluR1/5-mediated excitatory currents. Additionally, G protein-independent mechanisms primarily involve recruitment of β-arrestin, which mediates effectors anchoring and activation of other signaling pathways, like phosphatidylinositol 3-kinase/Akt/mammalian target of rapamycin (PI3K-Akt-mTOR) kinase pathway, which affects protein synthesis, cell growth and survival. Homer 1b/c isoforms allow formation of multimolecular protein complexes, which bridge mGluR1/5 with various intracellular effectors or ligand-gated receptors, like NMDARs, while Homer 1a isoforms antagonize Homer 1b/c interactions, thus affecting mGluR1/5 signaling and functions.

Canonical mGluRI signaling is mediated by G_q_/_11_-activated pathways, mainly resulting in the activation of phospholipase C β (PLCβ), which mediates the hydrolysis of phosphatidylinositol and generation of inositol-1,4,5-trisphosphate (IP_3_) and diacylglycerol (DAG), thus leading to Ca^2+^ intracellular mobilization from internal stores and activation of protein kinase C (PKC) [2]. Besides G_q_/_11_-dependent signaling, additional pathways downstream to mGluRI activation include either G_i/o_- or G_s_-mediated pathways, as well as other G protein-independent mechanisms, which requires the binding of β-arrestin, favored by receptor phosphorylation by G protein-coupled receptor kinases (GRKs) [3]. Overall, mGluRI stimulation can activate a list of effectors, including phospholipase D (PLD), protein kinases pathways such as casein kinase 1 (CK1), cyclin-dependent protein kinase 5 (CDK5), components of the family of the mitogen-activated protein kinases (MAPK), like extracellular signal-regulated kinase (ERK) and c-Jun N-terminal kinase (JNK), as well as phosphatidylinositol 3-kinase-Akt-mammalian target of rapamycin (PI3K-Akt-mTOR) kinases signaling pathway [4] (Figure 1). G protein-dependent and -independent mechanisms, downstream to mGluRI stimulation, can either result in the stimulation of distinct signaling pathways or converge in the activation of similar ones, as occurring for ERK, which can be activated transiently by PKC, or more persistently, through β-arrestin-dependent mechanisms [5,6].

mGluR1 and mGluR5 are functional as homodimers. Additionally, they can form intra-group heterodimers, mGluR1-mGluR5, but cannot associate with other mGluRs belonging to group II and III [3]. mGluR1 and mGluR5 display widespread and similar brain distribution, being either co-expressed in the same neuronal subtypes, although not always to the same degree of expression, or having a more segregated localization in distinct cellular populations [3,4]. In light of this, and because of their high sequence homology and shared signaling pathways, mGluR1 and mGluR5 have been classically considered as interchangeable, achieving redundant functions. However, they can either have separate roles and preferential expression in distinct cellular types, or be cross-talking, by acting sometimes in a cooperative otherwise antagonistic manner in the same neuronal population [3,4,7,8,9].

Regarding the subcellular localization, both mGluR1 and mGluR5 are mainly postsynaptic, localized at the edge of the postsynaptic density (PSD) in perisynaptic zones, where they interact with different scaffolding proteins to form a multiprotein signaling complex with downstream intracellular effectors and other ligand-gated receptors and/or ion channels. This organization is essential for their main function—the modulation of excitatory neurotransmission—since protein interactome regulates either mGluRI surface expression or the efficacy of intracellular signaling transduction [3,10,11,12,13]. To date, they have been identified several proteins interacting with mGluR1 and mGluR5. An important group of scaffolding proteins is represented by the family of Homer proteins, which include long isoforms (Homer 1b/c) able to form multimeric complexes, and a shorter isoform (Homer 1a), which cannot form multimeric complexes, and antagonize Homer longer isoforms connections [13,14]. Homer longer isoforms link mGluR1 or mGluR5 and their principal signaling effectors, like PLC, PI3K and the PI3K enhancer, PIKE-L, as well as the IP_3_ receptor, located on endoplasmic reticulum membrane, and transient receptor potential channels (TRPCs) [15,16,17]. Such connections occur by binding of their PDZ and EVH-1 domains to a proline-rich domain present in the C-terminal tail of mGluR1 and mGluR5. Homer long isoforms, by connection with other scaffolding proteins like PSD-95 and Shank, provide direct connection between mGluRI and other integral membrane proteins, including NMDARs, by physical interaction with NR2 subunits [18]. Besides Homer family, several other mGluR1- and mGluR5-binding proteins have been identified, such as other scaffolding proteins like tamalin, the neuron-specific protein norbin, the Ca^2+^-modulated protein calmodulin, the ubiquitin ligase Siah-1A, NECAB2, CAIN, various protein kinases, including PKC, GRK2, CaMKII, and cytoskeletal components, like the cytoskeletal protein 4.1 G [2,3,4,12,13].

mGluR1 and mGluR5 also bind various regulatory proteins, including members of the family of GRKs (GRK2 and GRK3 for mGluR5 and GRK2, GRK4 and GRK5 for mGluR1), which control, by phosphorylation, mGluRI internalization [19,20,21,22], as well as components of the family of the Regulator of G-protein Signaling (RGS), like RGS-4, which by increasing GTPase activity of Gα_q_ lead to uncoupling with G protein-linked effectors, and switch-off of mGluRI signaling [23]. Besides them, other regulatory proteins can interact with intracellular domains of mGluR1 and mGluR5, thus influencing mGluRI membrane docking and signaling properties.

mGluRI can also constitute protein complexes, by assembly with other GPCRs or ligand-gated receptors. There is evidence for mGluRI crosstalk with adenosine (A)-, dopamine (DA)-, GABAergic- and ionotropic glutamate receptors, resulting in dimeric- or trimeric interplay, like in the case of mGluR1-A_1_, mGluR1-NMDAR, mGluR1-GABA_B_, or mGluR5-A_2A_-D2 and mGluR5-A_2A_-NMDAR [24,25,26,27,28]. Such crosstalk with other receptors confers additional complexity to signaling pathways/intracellular mechanisms possibly linked to mGluRI stimulation. Therefore, ultimately, it is the mGluRI interactome, consisting of their binding with intracellular scaffolding proteins, regulatory proteins and signaling effectors, as well as their assembly with other receptors/ion channels, which definitively shapes factual mGluRI functions in different contexts, cellular populations, and brain areas.

### Protein Kinases-Dependent Regulation of mGluRI: Focus on Tyrosine Kinases

Protein phosphorylation is a post-translational modification decisive for the control of protein expression and function. It is mediated by specialized enzymes, called protein kinases, which catalyze the transfer of a phosphate group from ATP to determinate aminoacids (serine, threonine, or tyrosine) of the substrate proteins. Protein kinases are classified in serine/threonine- or tyrosine kinases, based on the targeted aminoacidic residues, and can be either intracellular diffusible proteins or integral membrane proteins, represented by the classes of receptor serine/threonine receptor kinases (RSTKs) and receptors tyrosine kinases (RTKs).

**Figure 2:**
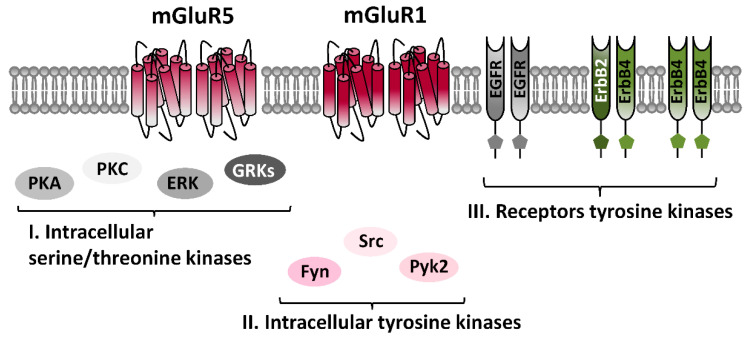
mGluRI interaction with protein kinases. Scheme illustrating protein kinases targeting mGluR1 and mGluR5; they include members of the families of (I) intracellular serine/threonine kinases like G protein-coupled receptors kinases (GRKs), protein kinase A (PKA), protein kinase C (PKC) and extracellular signal-regulated kinases (ERK), as well as (II) intracellular tyrosine kinases, such as Src, Pyk2, and Fyn, and (III) receptors tyrosine kinases, like the epidermal growth factor receptor (EGFR) and avian erythroblastic leukemia viral oncogene homologs (ErbB) receptors, ErbB2-4.

As many receptors, mGluR1 and mGluR5 are subjected to an active endogenous cycle of phosphorylations/dephosphorylations; such events deeply influence mGluR1/5 trafficking, subcellular distribution, and coupling with intracellular signaling effectors, thus being essential to proper mGluR1/5 function [29,30]. Some information on responsible kinases and the mechanisms underlying their modulatory roles is emerging, but the general picture describing kinases-mediated regulation of mGluR1/5 is probably still partial. It is known that mGluR1/5 are substrates of various intracellular serine/threonine kinases (Figure 2). Functional consequences of such phosphorylations are various, impacting either the efficacy of coupling with effectors, or retrieval from membrane surface [29,30,31,32]. Among serine/threonine kinases regulating mGluR1/5 activity are included some members of the family of GRKs, like GRK2, GRK3, GRK4, and GRK5. GRKs specifically recognize and phosphorylate agonist-activated GPCR, and foster GPCR desensitization. Thus, the GRKs-mediated mGluR1/5 phosphorylations represent a direct regulatory mechanism to limit enduring mGluR1/5 activation [19,20,21,22]. Other serine/threonine kinases modulating mGluR1/5 are second-messengers activated kinases, like protein kinase A (PKA), PKC, and ERK, that directly interact with the C terminal domain of mGluR1/5 [29,30,31,32]. PKA-dependent mGluR1/5 phosphorylations appear to regulate specific signaling pathway of mGluRI, as supported by the evidence that the mGluRI-dependent ERK activation is prevented by the inhibition of PKA [33,34]. Diverse PKC-dependent phosphorylations have been identified on either mGluR1 and mGluR5; such PKC-dependent mGluRI phosphorylations mainly foster receptor internalization [32,35,36]. ERK-dependent phosphorylation of mGluR1/5 occurs at the Homer binding site, and is involved in the modulation of either mGluRI surface expression and signaling [31,32].

While the involvement of various serine/threonine kinases in the modulation of mGluR1/5 has been better identified, the role of tyrosine kinases (TKs) in mGluRI regulation is less acknowledged. Nonetheless, some biochemical and electrophysiological evidence demonstrate that TKs activity is central for mGluRI functioning. In an earlier investigation it has been reported that tyrosine phosphorylation is instrumental for mGluR1 coupling to protein G_q/11_, and consequently, it affects G_q/11-_activated signaling pathways, downstream to mGluR1 [37]. Analyses of tyrosine phosphorylation of mGluRI then revealed that mGluR5 is tyrosine phosphorylated in basal conditions in the striatum, hippocampus, and cortex in rat brain [38]. Ratio of such tyrosine phosphorylation is increased by inhibition of tyrosine phosphatase, thus suggesting that mGluR5 are subjected to active cycles of phosphorylation/dephosphorylation [38]. mGluR1 in the striatum appears less phosphorylated in tyrosine residues in basal conditions [38], but the analysis of mGluR1 phospho-tyrosine ratio has not been extended to other brain areas. 

Despite accurate tyrosine phosphorylation sites on mGluR1 and mGluR5 have yet to be mapped in detail, the contribution of distinct TKs in mGluRI phosphorylation is emerging, along with the appreciation of their important roles in the regulation of mGluRI functions [39] (Figure 2). TKs can be primarily classified in a) non-receptor TKs, which are intracellular diffusible kinases, like the prototypical Src family, that include nine members: Src, Yes, Fyn, and Fgr, forming the SrcA subfamily, Lck, Hck, Blk, and Lyn in the SrcB subfamily, and Frk in its own subfamily, and b) receptor TKs (RTKs), subdivided in 20 subfamilies, which comprise receptors of growth factors and hormones, like nerve growth factor (NGF), brain-derived neurotrophic factor (BDNF), insulin, epidermal growth factor (EGF), and neuregulins (NRGs) [40].

The factual relevance of the TKs-dependent phosphorylation on mGluRI activity began to appear following investigations of mGluRI’s effects during pharmacological TKs inhibition. Earlier studies with non-specific TKs inhibitors, like genistein, have revealed that mGluR1-activated currents require an intact TKs activity, as observed in diverse neuronal populations/brain areas, including hippocampal CA3 pyramidal neurons [41], midbrain dopaminergic (DAergic) neurons of substantia nigra pars compacta (SNpc) and ventral tegmental area (VTA) [42], and cerebellar GABAergic interneurons [43]. Additional mGluRI functions, besides mGluR1-activated currents, are sensitive to a broad TKs inhibition with genistein, including mGluRI-dependent intracellular Ca^2+^ mobilization [37,42] and mGluRI-increased excitability leading to epileptiform bursts insurgence in cortical neurons [44]. Either brain area- or neuronal population-related differences have been reported in the TKs-dependent mGluRI regulation unmasked by broad TKs inhibitors, since in the cerebellum TKs negatively modulates mGluR1-mediated excitatory postsynaptic currents (mGluR1-EPSCs) at parallel fibers to Purkinje neurons (PF-PN) synapses [45], while TKs activity is instead required for proper mGluR1-induced currents in cerebellar GABAergic interneurons [43].

Additional insights on the functional relevance of the TKs-dependent phosphorylation of mGluRI arose following functional studies with more selective drugs, that specifically target subclasses and/or single TKs, as well as through the investigation of phosphorylated tyrosine sites on mGluR1 and mGluR5 intracellular domains, serving as TKs interacting sites. Various non-receptor TKs are expressed in neurons of mammalian brain, with some proteins, like members of Src family kinase, like Src and Fyn, being particularly expressed at synaptic sites, and thus more studied for potential roles in the regulation of synaptic activity and plasticity [39]. It has been reported that Src contributes to the regulation of mGluR1 signaling and function. Indeed, Src inhibition reduces mGluR1-activated inward currents in hippocampal CA3 pyramidal neurons [41] and dampens mGluR1-dependent activation of ERK2 [46]. mGluR1 is also a target of Fyn, which is directly bound to its intracellular C terminal domain [47]. Fyn-mediated phosphorylation of mGluR1a is constitutively active and facilitates its surface expression and function in rat cerebellar neurons [47]. Thus, Fyn-mediated phosphorylation positively controls mGluR1 activity.

Other evidence supports a functional interplay between mGluRI and another non-receptor TKs, the proline-rich tyrosine kinase 2 (Pyk2). Pyk2 interacts with both mGluR1 and mGluR5 through their second intracellular loop and carboxyl-terminal tail domains, and endogenous complexes of Pyk2 with mGluR1 have been detected in rat cerebellum brain homogenates [48]. The functional effect of the Py2k-induced phosphorylation of mGluRI appears multifaceted, since in heterologous expression systems Pyk2 attenuates mGluR1a signaling, by impairing its association with G protein Gα_q/11_, whereas in cortical neurons Py2k fosters mGluR1-induced ERK1/2 activation. Thus, Pyk2-dependent phosphorylation might affect mGluR1 signaling, possibly favoring activation of discrete signaling pathways [47].

Concerning RTKs roles in mGluRI modulation, it has been demonstrated a reciprocal functional crosstalk between mGluR5 and the epidermal growth factor receptor (EGFR). Indeed, mGluR5 stimulation induces EGFR transactivation, that promotes its physical association with mGluR5, and on the other side, EGFR inhibition, either pharmacological or genetic, reduces mGluR5 functions, like the mGluR5-stimulated Ca^2+^ signaling [46,49,50]. Additionally, in midbrain DA neurons, mGluR1-activated currents and mGluR1-induced intracellular Ca^2+^ mobilization are impaired by a TKs inhibitor, tyrphostin B-52, that inhibits EGFR, and few other TKs including avian erythroblastic leukemia viral oncogene homolog 2 (ErbB2) [42]. More recently, additional insights on the functional relevance of the RTKs on mGluRI have been provided from studies from our group proving that proper mGluRI function relies on an intact basal activity of RTKs of ErbB family, activated by the neurotrophic factors neuregulins (NRGs) [51,52,53,54]. As will be discussed below, after an introductive paragraph on ErbB receptors and their ligands, mGluRI activity is tightly dependent on ErbB activity, and the functional interaction between mGluRI and ErbB has various implications in the regulation of excitability, neurotransmission, synaptic plasticity, and learning processes [51,52,53,54].

## 2. ErbB Receptors and Their Ligands

**Figure 3:**
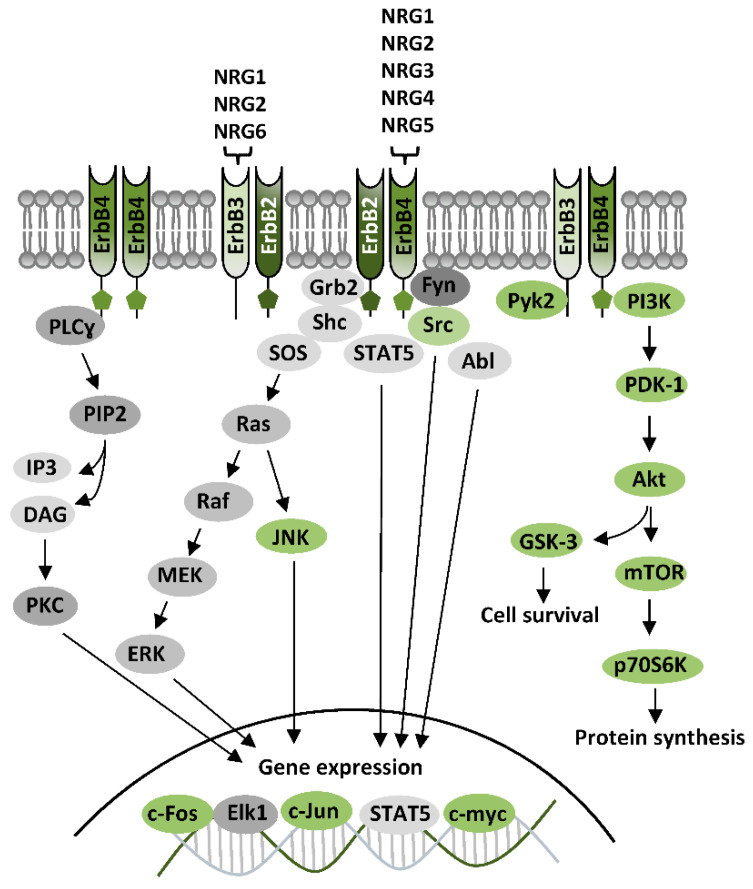
ErbB receptors and their signaling pathways. Diagram showing functional ErbB dimers, neuregulins (NRGs) binding and canonical ErbB signaling pathways. NRGs-induced ErbB stimulation activates PI3K-Akt-mTOR and GSK-3 pathways, which foster protein synthesis and cell survival, as well as Ras-Raf-MEK-ERK pathway or PLC-PKC pathway, mainly involved in gene expression regulation. Other ErbB effectors are kinases like Src, Abl, Pyk2, Fyn, JNK, and the signal transducer and activator of transcription 5 (STAT5), which affect gene expression by regulation of nuclear transcriptional factors.

The family of ErbB receptors constitutes the Class I of RTKs and is composed by four subtypes (ErbB1-4). ErbB1 is the epidermal growth factor (EGF) receptor (EGFR), whereas ErbB2, ErbB3, and ErbB4 are receptors of NRGs. ErbB receptors are functional as dimers, and distinct ErbB subtypes can differently participate in the formation of homodimeric or heterodimeric complexes, based on their distinctive properties, having each ErbB subtype a unique profile in relation to ligand binding properties and affinities, or catalytic activity [54,55,56]. Ligands for ErbB1—the EGF family ligands—are, besides EGF, transforming growth factor alpha (TGFα), heparin-binding EGF-like growth factor (HB-EGF), amphiregulin (AR), epiregulin (EPR5), betacellulin, (BTC), and epigen (EPG). The family of NRGs includes various components: NRG1 (also known as heregulin (HRG), Neu differentiation factor (NDF), acetylcholine receptor-inducing activity (ARIA), glial growth factor (GGF), or sensory and motor neuron-derived factor (SMDF)), NRG2 (also called NTAK), NRG3, NRG4, NRG5 (also known as tomoregulin), and NRG6 (also known as neuroglycan C) [54,55,56,57]. Different NRGs types display diverse affinity for ErbB3 and ErbB4, while none directly bind ErbB2, which is thus an indirect NRGs receptor, indirectly activated by NRGs through the others NRGs-binding subunits (ErbB3 and ErbB4). The features of distinct ErbB subunits (i.e., the presence of ligand-binding sites and/or of the active catalytic domains) dominate subunit assembly in the formation of ErbBs homo- and/or heterodimers. Amongst NRGs receptors, ErbB4 is the only autonomous subunit, because it has both NRGs-binding sites and active kinase domains, hence it is the sole able to form both homodimers and heterodimers. ErbB3 has ligand-binding sites, but does not have an active kinase domain, thus, it cannot form homodimers, nor directly phosphorylate other ErbB subunits, but can associate with ErbB2 or ErbB4 forming heterodimers [55]. ErbB2, otherwise, has an active kinase domain, but does not bind NRGs or other identified ligands (is still an orphan receptor). Nevertheless, ErbB2 is the preferred dimerization partner among all ErbB subunits [58], and dimerization with ErbB2 potentiates NRGs’ binding affinity for ErbB3 and ErbB4 [59,60]. Overall, NRGs effects can be mediated by ErbB4-ErbB4 homodimers, or ErbB2-ErbB4, ErbB2-ErbB3, and ErbB3-ErbB4 heterodimers, with distinct NRGs having specific binding affinities for diverse ErbB subunits, as NRG1 and NRG2 bind both ErbB3 and ErbB4, whereas NRG3, NRG4, and NRG5 only bind to ErbB4 and NRG6 binds to ErbB3 (Figure 3).

### ErbB Signaling

First step of ErbB activation—the ErbB-ErbB dimer formation—is induced by NRGs binding, which drives a conformational changes of ErbB subunits, fostering their trans-phosphorylation and the consequent recruitment of proteins having phosphotyrosine binding sites for Src homology-2 (SH2) domains, i.e. proteins acting as adaptors or effectors of ErbB signaling pathways. Typical ErbB-activated pathways are PI3K-Akt-mTOR, Ras-Raf-MEK-ERK, and PLC-PKC, as well as kinases like c-Abl, JNK, CDK5, Lyn, Pyk2, and glycogen synthase kinase-3 (GSK-3) [54,56,61] (Figure 3). The activation of PI3K-Akt-mTOR, and GSK-3, downstream to Akt, has been associated to NRGs/ErbB-dependent mechanisms that initiate protein synthesis and cause neuronal growth and survival. The stimulation of Ras-Raf-MEK-ERK requires the recruitment of the adaptor protein, the growth factor receptor/bound protein 2 (GRB2), which binds ErbB subunits, either directly or through the adaptor protein Shc. ErbB-GRB2 then activates Son of Sevenless (SOS), a guanine nucleotide exchange factor, which fosters Ras activation, directly inducing cascade stimulation of Raf, MEK, and ERK. Phosphorylated ERK translocates to the nucleus, where it activates transcriptional factors like Elk1, hence promoting transcription of genes regulating cell growth and survival. ERK also phosphorylates cytoskeletal proteins, like actin, which promote cell motility, or regulators of cell division and organelle movement, as well as mitochondrial targets such as Bcl2 that render cells resistant to apoptosis. Other NRGs/ErbB-activated pathways, like the PLC-PKC or c-Abl, JNK, CDK5, Lyn, and Pyk2, are mainly involved in gene expression regulation, by activation of transcriptional factors like c-Fos, Elk1, STAT, c-Jun, and c-Myc [54,56,61] (Figure 3).

Besides canonical ErbB signaling, elicited by NRGs binding, there are other NRGs/ErbB signaling modalities, namely “non-canonical forward ErbB signaling” and “NRG1 backward signaling”. Such signaling modalities require a proteolytic cleavage, which can occur on ErbB4 or on membrane-anchored NRGs, with the release of intracellular domains that translocates to the nucleus and activates gene transcription, [54,56,61]. Thus, NRGs/ErbB signals can be transmitted either in forward (canonical and non-canonical) and backward modalities, with the canonical ErbB signaling (Figure 3) being per se associated to a complex network of intracellular pathways.

## 3. ErbB-Dependent Regulation of mGluRI

### 3.1. ErbB-Dependent Modulation of mGluRI: Mechanisms

**Figure 4:**
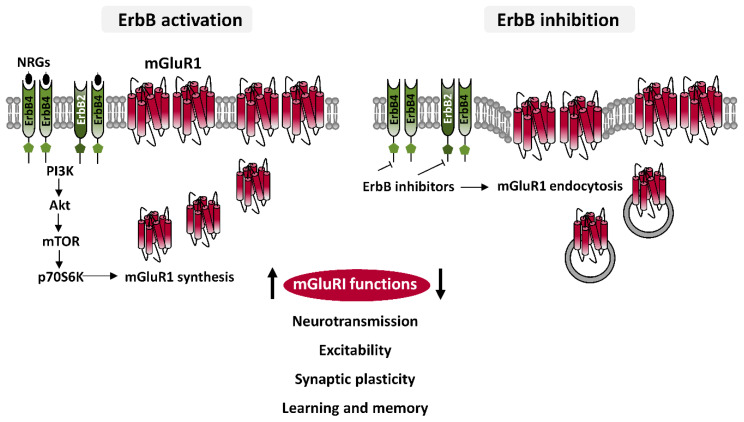
ErbB-dependent regulation of mGluRI. Diagram illustrating cellular mechanisms underlying ErbB-dependent regulation of mGluR1 showing that ErbB stimulation, by PI3K-Akt-mTOR pathway, induces mGluR1 synthesis and their trafficking to membrane, thus enhancing mGluR1 functions, whereas ErbB inhibition causes mGluR1 endocytosis, thus impairing mGluR1 activity. Pharmacological ErbB modulation hence can regulate different mGluRI-dependent effects, affecting neuronal depolarization, excitability, glutamatergic synaptic plasticity, learning and memory processes, as object recognition memory, and modulating in vivo activation of dopaminergic nigrostriatal pathway.

Investigations on the subcellular localization of ErbB4, the most studied ErbB subunit, have revealed their preferential localization in the glutamatergic postsynaptic densities (PSD), where they interact with PSD-95 [56,62,63], which is an important scaffolding hub leading glutamatergic post-synapse architecture, by docking glutamatergic receptors, both ionotropic NMDAR and AMPARs, or mGluRs, mainly mGluRI, directly or by association to other proteins. ErbB2 are also localized in the glutamatergic PSD, wherein they interact indirectly with PSD-95, through Erbin, a protein essential for ErbB2 membrane docking and function [64,65]. While it appears evident that ErbBs are well-positioned to physically interact, directly or indirectly, with mGluRI, a dedicate investigation on the physical mGluRI-ErbB assembly is nowadays missing. Nevertheless, insights on their functional crosstalk and on the cellular/subcellular sites of mGluRI-ErbB interaction have been provided by cell-confined functional analyses of mGluRI during pharmacological ErbB modulation. Studies from our groups have demonstrate that ErbB receptors bidirectionally control mGluRI: NRGs-induced ErbB stimulation potentiates mGluRI functions, whereas inhibition of tonic ErbB activity reduces mGluRI effects [51,52,53,54] (Figure 4). Cellular mechanisms underlying such potent ErbB-dependent control of mGluRI have been partially elucidated, mainly following an investigation of NRGs/ErbB-induced regulation of mGluR1 subtype in rat midbrain DA neurons, that has demonstrated that basal ErbB signaling is required for mGluR1 docking to cellular membrane, conferring maintenance of receptor surface level and function [51]. Indeed, pharmacological ErbB inhibition, rapidly causes mGluR1 endocytosis in midbrain DA nuclei, and a parallel impairment of mGluR1-activated inward currents in DA neurons, which is prevented by counteracting dynamin-dependent mGluR1 endocytosis [51].

In addition to preserve proper mGluR1 surface exposition, ErbB receptors are actively involved in the regulation of expression levels of mGluR1, by a direct regulation of their synthesis [51]. ErbB stimulation, with NRG1, increases mGluR1 protein levels in rodent midbrain DA nuclei and mGluR1 immunolabeling in single DA cells. NRG1-activated mGluR1 synthesis is promptly induced, being temporally preceded by activation of ErbB4 and PI3K-Akt-mTOR kinases pathway, a signaling pathway typically fostering protein translation [51]. Neo-synthesized mGluR1 are quickly distributed to surface membrane of midbrain DA neurons, and efficiently associate with their intracellular effectors, as proven by potentiated mGluR1 function (i.e., mGluR1-mediated inward currents) in single DA neurons following NRG1-induced ErbB activation. Such an increase in mGluR1-mediated inward currents in DA cells, by ErbB stimulation is prevented by protein synthesis inhibition, with drugs like anisomycin or cycloheximide [51]. While, based on brain distribution of ErbB subunits, multiple ErbB dimers could contribute to such regulation, cumulative evidence obtained from biochemical and functional analysis points to ErbB4-ErbB2 dimers as the major player involved in mGluR1 modulation. In addition to this dimer, other receptor pools, in particular ErbB4-ErbB4 homodimers, could be also overlapping contributors to mGluR1 regulation. It will be interesting to investigate if discrete ErbB dimers predominate in the control of mGluR1 and mGluR5 functions in different brain areas or cellular populations, as well as in the activation of discrete mGluRI signaling pathways. To this regard, an earlier report documented that mGluRI-dependent ERK2 activation is regulated by ErbB1, but not ErbB2, in rat astrocytes cultures [46], whereas the contribution of ErbB2 is clearly demonstrated in the regulation of various mGluRI functions either in rat midbrain DA neurons or in mouse hippocampal CA1 pyramidal cells [51,52,53].

Overall, the current picture indicates that ErbB receptors can control mGluR1 by directly affecting various steps of their functional lifecycle, from synthesis and distribution to membrane, to internalization. In conclusion, ErbB receptors can be recognized as vital partners in shaping mGluRI activity, with the implication that ErbB tone can deeply influence, through mGluRI, important neuronal and brain functions.

### 3.2. ErbB-Dependent Regulation of mGluRI: Functional Implications

Which is the functional relevance of the interaction between mGluRI and ErbB receptors? The essential role of ErbB signaling in sustaining mGluRI activity has been revealed by studies proving impaired mGluRI functions in presence of ErbB inhibitors, studies that unmasked ErbB influences on important neuronal and brain functions, like the control of neuronal excitability, neurotransmission and synaptic plasticity, besides their contribution in learning processes, which also involve mGluRI [51,52,53,54]. To date, such crosstalk between ErbB and mGluRI has been reported in the midbrain DA nucleus, SNpc, and in the hippocampus CA1 area.

#### 3.2.1. ErbB-mGluRI Interaction in Neuronal Depolarization and Excitability

mGluRI can affect neuronal excitability by a direct modulation of ion channels, or by a crosstalk with other ligand-gated ion channels. mGluR1 and mGluR5 mostly induces depolarizing currents, by opening cationic TRPCs [42,66,67], or by inhibiting K^+^ channels [68], but their activation can also produce hyperpolarization, by opening Ca^2+^-activated K^+^ channels [69]. Additionally, mGluRI can affect neuronal excitability by modulating other ligand-gated ion-channels, like NMDARs. While the net resultant of mGluRI activation (if excitatory or inhibitory) can be context-dependent or associated to subtype/duration of its stimulation, in most neurons mGluRI activation is excitatory, increases neuronal excitability and foster bursts firing generation [51,53,66,67,70,71].

In midbrain DA neurons, mGluRI agonists induce TRPCs-mediated inward currents, which are mainly dependent on mGluR1 activation, with a minor mGluR5 contribution [42,51,66]. ErbB activation, with NRG1, potentiates such mGluR1-activated currents in midbrain DA neurons, by directly inducing synthesis of mGluR1, which translocate to membrane and efficiently couple with conductance effectors [51]. Protein synthesis inhibitors indeed prevent such ErbB-elicited increase in mGluR1-mediated currents [51]. Contrariwise, inhibition of basal ErbB signaling (with pan-ErbB- or specific ErbB2 inhibitors) impairs mGluR1-activated currents, because it causes mGluR1 endocytosis inside DA neurons [51]. 

In hippocampal CA1 pyramidal neurons, mGluRI activation also regulates cellular excitability, by inducing neuronal depolarization and inward currents [53,70]. ErbB activity appears vital also for such mGluRI function, by bidirectionally controlling mGluRI-induces increase in excitability [53], similarly to midbrain DA neurons. Indeed, pharmacological ErbB inhibition or stimulation is able to dampen or potentiate, respectively, mGluRI-increased excitability of mouse hippocampal CA1 pyramidal neurons [53].

#### 3.2.2. ErbB-mGluRI Interaction in Glutamatergic Synaptic Plasticity

mGluRI activation causes a long term depression (LTD) of glutamatergic synaptic transmission in several brain areas, including hippocampus, dorsal and ventral striatum, medial prefrontal cortex, cerebellum, and midbrain DA nuclei [72]. Evidence from our group demonstrates that ErbB receptors are instrumental to such forms of mGluRI-dependent synaptic plasticity [54], either in the hippocampus at CA3-CA1 synapses [53] or in SNpc DA neurons [52]. 

mGluRI-dependent LTD at hippocampal CA3-CA1 synapses relies on mGluRI-induced AMPARs internalization, due to activation of several kinases, like ERK1/2, PI3K-Akt-mTOR, and MAPKs, which induces synthesis of proteins instrumental to LTD expression [73]. Hippocampal mGluRI-LTD is an underlying mechanism of learning and memory processes, and, its dysregulation has been reported in animal models of neurological and psychiatric disorders, including autism-spectrum disorders and genetic intellectual disabilities, as well as aging-related memory loss, Alzheimer’s disease, and schizophrenia [72,73,74,75,76,77,78,79,80,81]. Hippocampal mGluRI-LTD at CA3-CA1 synapses requires an intact basal ErbB activity [53,54]. Such ErbBs’ role in mGluRI-LTD expression has been revealed by using diverse ErbB inhibitors (broad-spectrum or selective ErbB2 targeting), all impairing mGluRI-LTD expression in CA1 pyramidal neurons from hippocampal mice slices [53,54]. On the other side, ErbB activation, by exogenous NRG1, facilitates LTD expression, in line with the ErbB-mediated bidirectional regulation of mGluRI, proved for other mGluRI functions.

mGluRI-dependent glutamatergic LTD has been also described in SNpc DA neurons, being due to reduced AMPAR-mediated synaptic transmission, consequent to selective mGluR1 activation [52]. ErbB receptors similarly control mGluR1-dependent LTD in SNpc DA neurons [52,54]. Basal ErbB activity is needed for proper mGluR1-dependent LTD expression, and, contrariwise, NRG1-dependent ErbB activation potentiates LTD magnitude and allows LTD induction during subthreshold/minimal mGluR1 activation [52,54]. ErbB2-ErbB4 dimers, expressed on DA neurons, are the best candidate involved in the regulation of mGluR1-dependent LTD, as shown by ErbB inhibition in single DA neurons with different drugs [52,54].

#### 3.2.3. ErbB-mGluRI Interaction in Learning and Memory Processes

Based on the evidence that basal ErbB signaling is required for hippocampal mGluRI-dependent LTD at CA3-CA1 synapses [53], our group has investigated ErbB’s role in a mGluRI-dependent learning process, i.e. object–recognition memory. mGluRI-dependent LTD in CA1 pyramidal neurons has been proposed as the biological substrate underlying novelty detection [72,73,82], mainly because it is endogenously induced in vivo in rodents during exploration of environments containing novel objects [82,83,84], and since object recognition is compromised when mGluRI-LTD expression at CA3-CA1 synapses is prevented [53,81,82,83,84,85,86].

In vivo hippocampal ErbB inhibition in CA1 area of mice impairs acquisition of novel object configurations, in parallel to impair mGluRI-dependent LTD at hippocampal CA3-CA1 synapses [53]. Thus, ErbB signaling acts as a gathering factor for proper hippocampal mGluRI-dependent synaptic plasticity and a related learning process [53,54].

#### 3.2.4. ErbB-mGluRI Interaction in the In Vivo Modulation of DA Release

mGluR1 activation directly contributes to modulate the state of activation of nigrostriatal pathway in vivo [51]. Indeed, intra-SNpc injection of a mGluRI agonist induces DA release in the projection areas in the dorsal striatum by activating nigral mGluR1 [51]. An intact basal ErbB tone is instrumental to this mGluR1-dependent activation of nigrostriatal pathway, as demonstrated by the evidence that intra-SNpc injection of a pan-ErbB inhibitor impairs mGluR1-induced striatal DA outflow [51]. ErbB2-ErbB4 are the best candidates possibly responsible for such mGluR1-induced nigrostriatal activation, because of this dimer involvement in the regulation of mGluR1-activated currents in DAergic neurons. Such ErbB-dependent modulation of mGluR1-controlled DA release implies that an unbalanced ErbB tone might affect midbrain DA neurotransmission.

### 3.3. ErbB-Dependent Regulation of mGluRI: Pathological Implications and Therapeutical Potential?

Currently it is still completely unknown if the NRGs/ErbB-dependent modulation of mGluRI is dysfunctional in pathological conditions, thus possibly contributing to the etiology of neurological and psychiatric disorders. Nevertheless, in light of the described functional implications of the ErbB-mediated mGluRI control, it appears evident that an abnormal ErbB activation, by profoundly affecting essential neuronal/brain functions, might feasibly participate to etiological mechanisms of neurological/psychiatric diseases in several ways. Even if the exact role of ErbB-mGluRI crosstalk in pathology will require dedicate investigations, in this context it is important to emphasize that, independently, either aberrant mGluRI functioning or altered NRGs/ErbB signaling have been repeatedly associated to various brain illnesses, including schizophrenia, bipolar disorder, autism spectrum disorders and genetic intellectual disabilities, as well as Alzheimer’s disease, major depressive disorder, Parkinson’s disease, and addiction [2,3,4,56,57,61,72,73,74,75,76,77,78,79,80,81,87,88,89,90,91,92,93,94,95,96,97,98,99,100]. Hence, investigating abnormal ErbB-dependent regulation of mGluRI in these brain illnesses might reveal a molecular mechanism linking pathological mGluRI and NRGs/ErbB alterations. Meanwhile, in light of the peculiar skill of ErbB in bidirectionally adjusting mGluRI function, it can be hypothesized that pharmacological ErbB targeting might be an approach to reestablish unbalanced mGluRI tone to adequate levels for proper neurotransmission, neuronal excitability and synaptic plasticity, thus possibly contributing to maintain normal cognitive functions and complex behaviors. In this viewpoint, ErbB might be probed as targets in the clinical conditions in which abnormal mGluRI function is a recognized pathological feature.

## 4. Conclusions and Open Issues

In conclusion, even if the general picture on the interplay between TKs and mGluRI is probably still partial, existing evidence supports their relevant role in the control of mGluRI. Current data mainly support a scenario in which tyrosine phosphorylation is required to preserve mGluRI membrane docking and signaling. Since G_q/11_ tyrosine phosphorylation fosters its coupling with mGluRI, G protein-dependent pathways would be more sensitive to TKs modulation.

Among different TKs, growing evidence identifies ErbB receptors as central modulators of mGluRI. ErbB activity tonically controls mGluRI surface expression levels, thus profoundly affecting core mGluRI functions. Indeed, mGluRI-dependent effects on neuronal excitability and synaptic plasticity, as well as mGluRI-regulated neurotransmitters release or aspects of mGluRI-modulated learning processes, are all disturbed by ErbB inhibition. On the other side, NRGs-induced ErbB stimulation, by increasing mGluRI total expression levels and trafficking to membrane, can strength various mGluRI functions.

Numerous open issues circumvent such functional interaction between mGluRI and ErbB receptors. Future studies, besides elucidating precise intracellular mechanisms and signaling pathways involved, might unveil if mGluRI-ErbB crosstalk varies among diverse brain areas/cellular populations, whether it is instrumental to proper cognitive functions and complex behaviors and, ultimately, if such ErbB-dependent regulation of mGluRI is disrupted in pathological conditions or ErbB targeting can be exploited to develop novel strategies for the treatment of brain disorders.

Of note, ErbB-dependent mGluRI modulation is an additional mechanism to consider in the main picture describing the functional impact of TKs on the control of essential brain functions, whose general view can be better appreciated when pondering that, besides mGluRI, several other TKs targets have been identified among ligand-gated ion channels or voltage-dependent channels, including glutamatergic NMDARs and AMPARs, GABAergic GABA_A_-, nicotinic acetylcholine receptors (nAChRs) and voltage-dependent ion channels [101,102,103,104], and all of them are chief players in the mechanisms sustaining neuronal excitability, neurotransmission and synaptic plasticity. Altered TKs activity might hence profoundly affect proper brain functions and feasibly contribute in several ways, including by causing aberrant mGluRI activity to the neurological bases of various brain diseases.
